# Epidemiologic Models, Key Logs, and Realizing the Promise of WHA 54.19

**DOI:** 10.1371/journal.pntd.0002092

**Published:** 2013-02-28

**Authors:** David G. Addiss

**Affiliations:** Children Without Worms, The Task Force for Global Health, Decatur, Georgia, United States of America; Swiss Tropical and Public Health Institute, Switzerland

During the late 1800s, when trees were cut by handsaw and the logs floated down river by the hundreds to be milled, they occasionally jammed. Skilled lumberjacks could often identify a single “key log," which, once released, opened the way for all the logs to continue their journey. The study by Anderson and colleagues in this issue of *PLOS Neglected Tropical Diseases* [1] points to the presence of such a key log. Using epidemiologic models, the authors address a straightforward question: if school-age children are regularly treated for soil-transmitted helminthiasis (STH), as recommended, can transmission be interrupted? In doing so, Anderson and colleagues open a fundamental conversation about the goals and expectations of current global STH control efforts.

Under the strong leadership of the World Health Organization (WHO), STH control has been gaining momentum since early studies showed the health and nutritional benefits of deworming in children [2]–[4]. In 2001, a World Health Assembly resolution (WHA 54.19) called for regular administration of preventive chemotherapy to at least 75% of school-age children at risk of STH morbidity. The primary goal was not interruption of transmission, which, in the absence of adequate water, sanitation, and hygiene education (WASH), is unrealistic, but rather, reduction in STH morbidity associated with high worm burdens. As with other neglected tropical disease (NTD) control programs, progress was limited, in part, by the cost of safe and effective drugs and the staggering number of people at risk—more than 2 billion [5], [6]. This situation changed dramatically in 2010 when Johnson & Johnson (J&J) increased its donation of mebendazole for STH from approximately 30 to 200 million doses per year and GlaxoSmithKline (GSK) pledged to donate up to 400 million doses of albendazole annually. Our challenge now is to use this unprecedented resource, some 5 billion doses between now and 2020, to achieve the greatest possible public health impact.

With very good reason, WHO has prioritized scaling up treatment for the estimated 615 million school-age children at risk of STH morbidity [6]–[8]. Intensity of infection with *Ascaris lumbricoides* and *Trichuris trichiura* typically peaks in this age group [9], and nutritional needs of school-age children for growth, development, and learning are substantial. Furthermore, schools have proven to be a highly practical and cost-effective platform for delivery of preventive chemotherapy [10]. Limiting the J&J and GSK drug donations to school-age children concentrates the benefits of deworming on this important and vulnerable population. Even with the convenience and efficiency of school-based distribution, the global effort required to provide preventive chemotherapy to 75% of school-age children at risk should not be underestimated—it is a mammoth undertaking.

Even so, too narrow a focus on a coverage target comes at a cost. First, 75% coverage alone is unlikely to achieve the morbidity reduction goal, which is operationalized as ≤1% prevalence of moderate and high-intensity infection [6]. Second, STH morbidity is not limited to school-age children, but also affects preschool-age children and women [6]. Many national STH control programs provide STH preventive chemotherapy to preschool children, as WHO recommends, often in combination with vaccination or vitamin A [6]. In 2010, for example, WHO reported that 37% of preschool children at risk for STH received treatment, compared to 28% of school-age children [8]. Women of childbearing age typically have higher intensity hookworm infection than school-age children and are particularly vulnerable to its associated anemia [11]. They are relatively neglected by STH control programs, although millions have received the deworming benefits of albendazole through the Global Programme to Eliminate Lymphatic Filariasis [12]. For the STH program manager, providing preventive chemotherapy to all three risk groups can be logistically and administratively complex. Drugs must be secured from different sources and delivered both through the schools and through different divisions of the health care system. This can require considerable savvy and inter-organizational cooperation.

Third, as the epidemiologic models of Anderson and colleagues indicate [1], even under the best of circumstances, treatment of school-age children alone is unlikely to have any lasting effect on transmission, especially for hookworm. Recrudescence of STH to pre-treatment levels is generally observed within a few years after treatment ends [13]. Without improvements in WASH to decrease transmission over the long term, regular deworming must be continued in perpetuity to achieve morbidity reduction goals. Thus, the study by Anderson and colleagues invites the loosely-knit STH control community into a conversation. What does “elimination of STH as a public health problem," as articulated in WHA 54.19, require between now and 2020? Are the intended beneficiaries primarily school-age children, or rather, all persons at risk of STH-related morbidity? Is STH control more correctly viewed as a component of school health or as an initiative to reduce STH morbidity and transmission in the community? Is increasing coverage in school-age children a critical first step toward comprehensive STH control or an end in itself?

Recently, the goals for controlling onchocerciasis and schistosomiasis, based in large part on years of experience and success with preventive chemotherapy, have shifted from morbidity control to the more ambitious interruption of transmission, at least at a regional level [14]. These shifts in emphasis were preceded by careful deliberation and much debate. Reducing transmission in the community is one of the aims of preventive chemotherapy for STH [8], yet the analysis by Anderson and colleagues indicates that the primary strategy, scaling up preventive chemotherapy coverage among school-age children, is unlikely, by itself, to realize this aim. Is it possible to maintain focus on the critically important need of scaling up treatment in school-age children while expanding our peripheral vision to include other risk groups and to implement social and environmental interventions that reduce transmission?

In addition to preventive chemotherapy, WHA 54.19 urges member states to “promote access to safe water, sanitation and health education through intersectoral collaboration." Even in the absence of preventive chemotherapy, sanitation may reduce prevalence and intensity of STH infection by approximately 50% [15]. Little is known about what combinations of drug coverage and WASH are required to realize a given impact on transmission. Validating relevant indicators for WASH and modeling the combined effects of WASH and preventive chemotherapy are important areas for further research.

Anderson and colleagues demonstrate the power of epidemiologic modeling to raise critical questions and sharpen our thinking. The key log that they reveal is the need for clarity of purpose and renewed commitment to the promise of WHA 54.19. From a clear, shared vision of elimination of STH as a public health problem, our strategies, objectives, and programs will naturally flow.

## References

[1] Anderson RM, Truscott JE, Pullan RL, Brooker SJ, Hollingsworth TD (2013) How effective is school-based deworming for the community-wide control of soil-transmitted helminths? PLoS Negl Trop Dis 7 (2) e2027 doi:10.1371/journal.pntd.0002027. 23469293 10.1371/journal.pntd.0002027 PMC3585037

[2] Adams E, Stephenson L, Latham M, Kinoti S (1994) Physical activity and growth of Kenyan school children with hookworm, Trichuris trichiura and Ascaris lumbricoides infections are improved after treatment with albendazole. J Nutrition 124: 1199–1206. 8074755 10.1093/jn/124.8.1199

[3] Stephenson LS, Latham MC, Adams EJ, Kinoti SN, Pertet A (1993) Physical fitness, growth and appetite of Kenyan school boys with hookworm, Trichuris trichiura and Ascaris lumbricoides infections are improved four months after a single dose of albendazole. J Nutrition 123: 1036–1046. 8505663 10.1093/jn/123.6.1036

[4] Stephenson LS, Latham MC, Adams EJ, Kinoti SN, Pertet A (1993) Weight gain of Kenyan school children infected with hookworm, Trichuris trichiura and Ascaris lumbricoides is improved following once- or twice-yearly treatment with albendazole. J Nutrition 123: 656–665. 8463866 10.1093/jn/123.4.656

[5] De Silva NR, Brooker S, Hotez PJ, Montresor A, Engels D, et al (2003) Soil-transmitted helminth infections: updating the global picture. Trends Parasitol 19: 547–551. 14642761 10.1016/j.pt.2003.10.002

[6] WHO (2011) Soil-transmitted helminthiases: eliminating soil-transmitted helminthiases as a public health problem in children: progress report 2001–2010 and strategic plan 2011–2020. Geneva: World Health Organization.

[7] WHO (2011) Helminth control in school-age children: a guide for managers of control programmes. 2nd ed. Geneva: World Health Organization.

[8] WHO (2012) Soil-transmitted helminthiasis: number of children treated in 2010. Wkly Epidemiol Rec 87: 225–232. 24340403

[9] Bundy DAP (1988) Population ecology of intestinal helminth infections in human communities. Phil Trans R Soc Lond B 321: 405–420. 2907151 10.1098/rstb.1988.0100

[10] Bundy D (2011) Rethinking school health: a key component of education for all. New York: The World Bank.

[11] Brooker S, Hotez PJ, Bundy DAP (2008) Hookworm-related anaemia among pregnant women: a systematic review. PloS Negl Trop Dis 2 (9) e291 doi:10.1371/journal.pntd.0000291. 18820740 10.1371/journal.pntd.0000291 PMC2553481

[12] WHO (2012) Global programme to eliminate lymphatic filariasis: progress report, 2011. Wkly Epidemiol Rec 87: 345–356. 22977953

[13] Jia T-W, Melville S, Utzinger J, King CH, Zhou X-N (2012) Soil-transmitted helminth reinfection after drug treatment: a systematic review and meta-analysis. PLoS Negl Trop Dis 6 (5) e1621: 10.1371/journal.pntd.0001621. 10.1371/journal.pntd.0001621 PMC3348161 22590656

[14] WHO (2012) Accelerating work to overcome the global impact of neglected tropical diseases: a roadmap for implementation. Geneva: World Health Organization.

[15] Ziegelbauer K, Speich B, Mäusezahl D, Bos R, Keiser J, et al (2012) Effect of sanitation on soil-transmitted helminth infection: systematic review and meta-analysis. PLoS Med 9 (1) e1001162 doi:10.1371/journal.pmed.1001162. 22291577 10.1371/journal.pmed.1001162 PMC3265535

